# 2041: Before hospice: Symptom burden, dementia, and social participation in the final years

**DOI:** 10.1017/cts.2017.91

**Published:** 2018-05-10

**Authors:** Halima Amjad, David Roth, Jennifer Wolff, Esther Oh, Quincy Samus

**Affiliations:** Johns Hopkins University, Baltimore, MD, USA

## Abstract

OBJECTIVES/SPECIFIC AIMS: Traditional hospice focuses on symptoms and quality of life (QOL) at the very end of life. Clinical symptoms and QOL in the last 1–2 years of life are also important and may be affected by dementia. Our objective was to characterize how symptoms differ between people with and without dementia in the last years before death and whether symptoms impact social dimensions of QOL. METHODS/STUDY POPULATION: We studied 1270 community-dwelling participants who died between 2011 and 2015 in the National Health and Aging Trends Study, a nationally representative cohort of older adults. From the last interview before death, we examined sensory (vision; hearing), physical (pain; problems with breathing, chewing/swallowing, speaking, upper or lower extremity strength/movement, and balance/coordination), and psychiatric (depression; anxiety; insomnia) symptoms by dementia status. We examined associations between symptoms and participation restrictions (visiting family/friends, attending religious services, participating in clubs/activities, going out for enjoyment, and engaging in favorite activity). RESULTS/ANTICIPATED RESULTS: Low energy (69%), pain (59%), and lower extremity strength/movement problems (56%) were most common. People with dementia (37.3% of decedents) had higher prevalence of all symptoms (*p*≤0.01), except pain, breathing problems, and insomnia. Dementia and greater symptom burden were independently associated with greater odds of participation restrictions (*p*<0.05). Problems speaking were significantly associated with limitations in all activities except for attending religious services. Balance/coordination, energy, and strength/movement problems were associated with limitations in 3 activities. DISCUSSION/SIGNIFICANCE OF IMPACT: Sensory, physical, and psychiatric symptoms are common in the year before death, with greater symptom prevalence in people with dementia. Both dementia and symptoms are associated with restrictions in participation. Older patients may benefit not only from earlier emphasis on palliative care but also programs and assistive devices that accommodate physical impairments.

